# Rheumatoid arthritis, as a clinical disease, but not rheumatoid arthritis-associated autoimmunity, is linked to cardiovascular events

**DOI:** 10.1186/s13075-022-02722-z

**Published:** 2022-02-24

**Authors:** Hélène Gouze, Philippe Aegerter, Roula Said-Nahal, Marie Zins, Marcel Goldberg, Guillaume Morelle, Georg Schett, Maxime Breban, Maria Antonietta D’Agostino

**Affiliations:** 1 Infection & Inflammation, UMR 1173, Inserm, UVSQ/Paris Saclay, 78180 Montigny-le-Bretonneux, France; 2 Service de Rhumatologie, Hôpital Ambroise Paré, AP-HP-Paris Saclay, 92100 Boulogne, France; 3 Laboratoire d’Excellence Inflamex, Université Paris Descartes, Sorbonne Paris Cité, Paris, France; 4 Inserm U1018 - Center for Research in Epidemiology and Population Health (CESP), Integrative Respiratory Epidemiology Team, Paris Saclay University, Villejuif, France; 5 Population-based Cohorts Unit-UMS 011, Paris University, Villejuif, France; 6 Department of Internal Medicine 3 - Rheumatology and Immunology, Friedrich-Alexander University (FAU), Erlangen-Nuremberg and Universitätsklinikum Erlangen, Erlangen, Germany; 7 Deutsches Zentrum für Immuntherapie, Friedrich-Alexander University (FAU), Erlangen-Nuremberg and Universitätsklinikum Erlangen, Erlangen, Germany; 8 Istituto di Reumatologia, Università Cattolica del Sacro Cuore, Fondazione Policlinico Universitario Agostino Gemelli IRCSS, Rome, Italy

**Keywords:** Rheumatoid arthritis, Cardiovascular risk, Cardiovascular diseases, Autoimmunity, Anti-citrullinated protein autoantibody

## Abstract

**Background:**

Rheumatoid arthritis (RA) is characterized by increased cardiovascular (CV) mortality. CV events are particularly high in patients with RA-specific autoimmunity, including rheumatoid factor (RF) and anti-citrullinated protein antibodies (ACPA), raising the question whether RA-specific autoimmunity itself is associated with CV events.

**Methods:**

New CV events (myocardial infarction, stroke or death by CV cause) were recorded in 20,625 subjects of the Electricité de France – Gaz de France (GAZEL) cohort. Self-reported RA cases in the GAZEL cohort were validated by phone interview on the basis of a specific questionnaire. In 1618 subjects, in whom plasma was available, RF and ACPA were measured. A piecewise exponential Poisson regression was used to analyze the association of CV events with presence of RA as well as RA-specific autoimmunity (without RA).

**Results:**

CV events in GAZEL were associated with age, male sex, smoking, hypertension, hyperlipidemia, and diabetes mellitus (HR from 1.06 to 1.87, *p* < 0.05). Forty-two confirmed RA cases were identified. Confirmed RA was significantly associated with CV risk increase (HR of 3.03; 95% CI: 1.13–8.11, *p* = 0.03) independently of conventional CV risk factors. One hundred seventy-eight subjects showed RF or ACPA positivity without presence of RA. CV events were not associated with ACPA positivity (HR: 1.52, 95% CI: 0.47–4.84, *p* = 0.48) or RF positivity (HR: 1.15, 95% CI: 0.55–2.40, *p* = 0.70) in the absence of RA.

**Conclusions:**

RA, as a clinical chronic inflammatory disease, but not mere positivity for RF or ACPA in the absence of clinical disease is associated with increased CV risk.

**Supplementary Information:**

The online version contains supplementary material available at 10.1186/s13075-022-02722-z.

## Introduction

Rheumatoid arthritis (RA) is an autoimmune chronic inflammatory disease characterized by synovitis leading to joint destruction and functional impairment [1]. Prevalence of RA is around 0.5% in Caucasian population [2]. RA development is promoted by a combination of genetic susceptibility and environmental factors that leads to breech of immune tolerance and formation of autoantibodies such as rheumatoid factor (RF) and anti-citrullinated peptide antibodies (ACPA) [1]. Autoimmunity precedes RA by several years (positive predictive value > 96% at 5 years) [3–5] and is associated with higher disease activity and structural damage [6, 7].

RA is characterized by an increased morbidity and mortality [8–11]. Besides structural damage and its consequences on disability, increased cardiovascular (CV) risk, including myocardial ischemia and heart failure, has been described in RA [10]. Aside from the consequences from disability and infections, CV disease is responsible for increased mortality in RA [8–11]. Increased CV risk in RA does not only seem to be explained by standard CV risk factors, but also by chronic inflammation, which accelerates the process of atherosclerosis [12, 13]. This process seems to be independent from the use of concomitant treatments such as corticosteroids or non-steroidal anti-inflammatory drugs (NSAIDs), which also add to CV risk [14].

In recent years, several studies suggested that RA with positive RF and/or ACPA presents a higher CV risk [9, 15–18]. Thus, one may think that RF and ACPA could influence CV risk independently from RA. However, evidence that CV disease may be linked to the presence of autoantibodies, independently from the occurrence of RA, is scarce, and only one study suggested that autoimmunity increases CV risk [15, 16, 19] in a subpopulation of African-American women, but not in all women with positive RF or ACPA [19]. To further explore this question, we made use of a large epidemiological cohort [20–22] and separately tested the influence of RA on CV risk as well as the impact of autoantibodies (RF/ACPA) on CV risk, independently of the presence of RA.

## Methods

### Study population

The GAZEL cohort was started in 1989 and included 20,625 current employees at that time of the French national company of services named “Electricité de France – Gaz de France” [20, 21]. Women were aged between 35 and 50 years and men between 40 and 50 years at inclusion, respectively. Demographic characteristics and a complete medical history were recorded in all subjects at baseline. Thereafter, subjects received an annual questionnaire covering information on a wide spectrum of pathologies, including rheumatic and musculoskeletal diseases (RMDs) as well as CV risk factors [20, 21]. In addition, plasma was collected from a fraction of the GAZEL cohort between 2000 and 2005. In 2010, a specific screening questionnaire dedicated to identify patients affected by inflammatory arthritis, including RA, was included in the GAZEL workup.

The GAZEL protocol was approved by the French authority for data confidentiality (‘Commission Nationale Informatique et Liberté’) and by the Ethics Evaluation Committee of the ‘Institut National de la Santé et de la Recherche Médicale (INSERM)’ (IRB0000388, FWA00005831).

### RA diagnosis ascertainment

All subjects who declared to suffer from RA in the 2010 screening questionnaire were included in the study. After having accepted to be contacted, patients were reached by phone and interviewed by an experienced rheumatologist trained for this purpose using a phone questionnaire specifically developed for ascertaining the diagnosis of RA (see Additional file 1). This questionnaire was previously validated on a panel of 102 consecutive outpatients consulting the rheumatology department of Ambroise Paré Hospital (Boulogne-Billancourt, France) for several RMDs (including RA, axial spondyloarthritis or psoriatic arthritis). The questionnaire was administrated by a physician blinded to the patients’ diagnosis, and its sensitivity and specificity for the diagnosis of RA were of 100% and 89%, respectively (see Additional file 1).

### ACPA and RF determination

Aliquots of plasma stored at − 80 °C were used to quantify the presence of ACPA and RF antibodies. Laboratory tests were realized in a specialized research laboratory (Department of Immunology and Internal Medicine, University of Erlangen-Nuremberg) and consisted in IgG ACPA ELISA (Reference Euroimmun EA 1505-9601 G) and IgM-RF ELISA (Reference IBL International RE70341). Cut-off value for ACPA was defined as positive if ≥ 4.6RU/mL, and for RF if ≥ 10 U/mL.

### Study timescale

GAZEL cohort began in 1989 with annual updates available for the statistical analysis since then. Blood sample collection was realized only between 2000 and 2005. The baseline year for our analysis was set at the end of the blood sample collection (2005). The number of patients with RA in the whole cohort was calculated using the self-reported diagnosis of RA collected through the questionnaire sent in 2010. These diagnoses were verified using the phone consultation, and we focused our analysis on the RA patients with a disease duration longer than 5 years, i.e., with a confirmed diagnosis of RA at the beginning of the analysis period (2005). Thus, RA diagnosis preceded the occurrence of new CV events. As blood samples were collected between 2000 and 2005 and death causes were available through 2014, the analysis on the occurrence of new CV events was made from the beginning of 2005 through 2014.

### Statistical analysis

Clinical and biological parameters (age, sex, CV risk factors, including high blood pressure, smoking habit, alcohol intake, obesity, diabetes, dyslipidemia, death), and the occurrence of CV events, including myocardial ischemia, non-lethal stroke, and death due to any CV cause were reported using descriptive statistics (mean and standard deviation (SD) or median and interquartile range). The outcome variable was the occurrence of new CV event, including non-lethal myocardial ischemia, non-lethal stroke or death due to CV disease. Non-lethal CV events were self-reported on the annual questionnaires. In these questionnaires, it was recommended to specify if the subject had suffered in the last 12 months of [1] myocardial infarction and [2] stroke. Lethal events were obtained by International Classification of Disease ICD-10 coding of death certificates. Codes of deaths due to CV cause concerned cardiac arrest, atherosclerosis, ischemic heart disease, chest pain, myocardial infarction, and all types of strokes. Explicative variables were multiple and concerned RA, autoantibodies (ACPA and RF) and already known CV risk factors. Cut-off values defining risk factors were considered as follows: presence of obesity if body mass index (BMI) ≥ 30, alcohol intake if ≥ 14 glasses per week for women and ≥ 21 glasses per week for men (according to World Health Organization (WHO) proposed cut-offs), and corresponding to moderate and high consumption according to the cut-offs proposed by the cohort team; tobacco consumption if the number of pack-years (PY) was ≥ 20, which corresponded to a consumption of 20 cigarettes a day for at least 20 years (and classified as moderate and heavy smoker by the cohort team). High blood pressure, dyslipidemia, and diabetes were also self-reported. Family history of myocardial infarction was considered when it occurred before the age of 60 years (mother) or 50 years (father). We used piecewise exponential Poisson regression, as the data were composed of discrete times of observation [23]. High blood pressure, dyslipidemia, diabetes, BMI, and tobacco and alcohol intakes were accounted as time-dependent covariates. Subjects who declared having RA but who were not reached by phone to confirm their diagnosis were excluded from the analyses. We made a comparison between subjects with and without available blood sample.

## Results

### Identification of CV events

GAZEL cohort enrolled 20,625 subjects, including 5614 women (27.2%) and 15,011 men (72.8%). Mean ± SD age at inclusion was 44.2 ± 3 years. Characteristics of the cohort at the beginning of the analysis period (2005) are reported in Table 1. From 2005 through 2014, a mean of 169 CV events occurred every year in the whole cohort. During this observation period, 1687 subjects in the whole cohort presented a new CV event, 129 of them were lethal and 1558 not lethal.

**Table 1:** Demographic and clinical characteristics of GAZEL subjects in 2005

Variable	Global cohort (***N*** = 19,557)	Subjects without auto-antibodies measurement (***N*** = 17,939)	Subjects with auto-antibodies measurement (***N*** = 1618)
Mean age (years)	60.2 ± 3.49	60.1 ± 3.51	60.6 ± 3.25^a^
Sex (% men)	14,149 (72.3%)	12,821 (71.5%)	1328 (82.1%)^a^
Mean retirement age (years)	55.3 ± 3	55.3 ± 3.1	55.4 ± 2.6
GAZEL 2005 questionnaire response rate	14,474 (74%)	12,906 (71.9%)	1568 (97%)^a^
**CV risk factors**			
Hypertension	3385 (17.3%)	3071 (17.1%)	314 (19.4%)^a^
Diabetes	799 (4.1%)	727 (4.1%)	72 (4.4%)
Hyperlipidemia	3999 (20.4%)	3559 (19.8%)	440 (27.2%)
Family history of myocardial infarction	1264 (6.5%)	1147 (6.4%)	117 (7.2%)
Body mass index	26 ± 3.7	26 ± 3.7	25.6 ± 3.3^a^
Smoking (% ≥ 20 pack-years)	4535 (23.2%)	4210 (23.4%)	325 (20.1%)
Alcohol consumption	2564 (13.1%)	2263 (12.6%)	301 (18.6%)
% ≥ to 21 glasses/week (men)			
% ≥ to 14 glasses/week (women)			
**CV events**			
All events	327 (1.7%)	306 (1.7%)	21 (1.3%)^a^
Stroke	98 (0.5%)	92 (0.5%)	6 (0.4%)
Myocardial infarction	229 (1.2%)	214 (1.2%)	15 (0.9%)

Results are expressed as mean (standard deviation) or number (percentage)

^a^Significant difference between groups (*p* < 0.05)

### Identification of RA patients

**Fig. 1:**
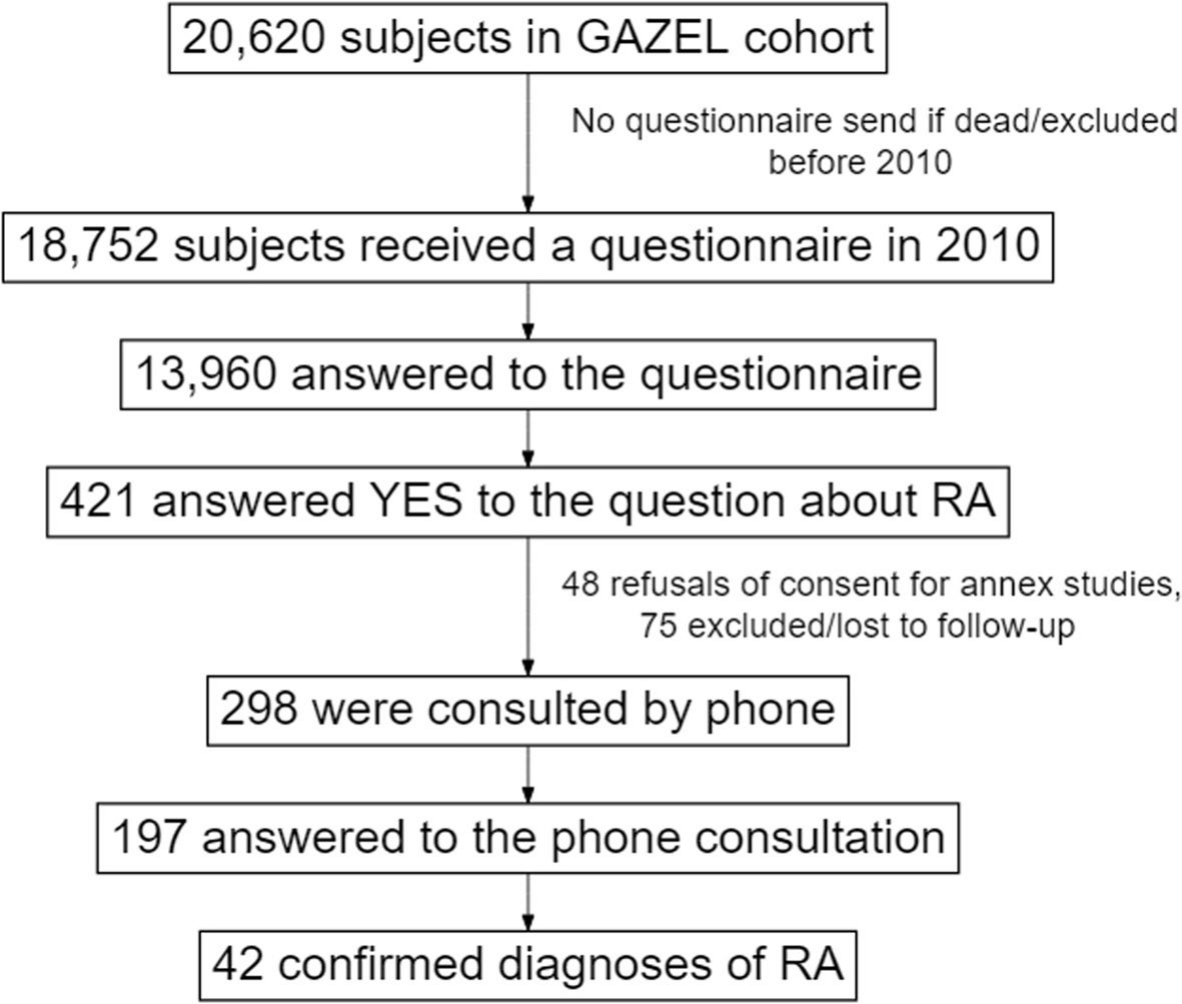
Flowchart of the rheumatoid arthritis (RA) diagnosis confirmation process

From the 18,752 subjects still followed in 2010, when the questionnaire on RMDs was administrated, 13,960 replied to that questionnaire. Four hundred twenty-one subjects declared themselves to have RA. Of these, 197 subjects were reached by phone and the RA diagnosis was confirmed in 42 of them and dismissed in 155 (see flowchart Fig. 1). Among the 42 confirmed RA, 30 were men and 12 were women with a mean ± SD age of 61.2 ± 3.4 years. Median RA duration was 9 years (range: 1–43 years). There was no significant difference at baseline (2005) between RA patients and the whole cohort.

Among the RA patients, 13 had plasma sample. Treatment information was available for 30 of them: 87% received at least one disease-modifying anti-rheumatic drug (DMARD) (26/30). Among them, 69% had only conventional synthetic DMARDs (18/26), 8% had only biological DMARDs (2/26) and 23% received both (6/26). Patients treated with corticosteroids represented 30% (9/30 patients), 78% of them took < 8 mg per day of prednisone.

### Association between RA and CV events

RA was significantly associated with an increased incidence of CV events in both univariable and multivariable analyses, with a hazard ratio (HR) of 3.03 (95% CI: 1.13–8.11, *p* = 0.03, multivariable analysis) (Table 2). CV events were also associated with established CV risk factors, such as male sex (HR: 1.87, 95% CI: 1.5–2.34, *p* < 0.001), tobacco consumption (HR: 1.54, 95% CI: 1.31–1.80, *p* < 0.001), high blood pressure (HR: 1.51, 95% CI: 1.30–1.76, *p* < 0.001), diabetes (HR: 1.25, 95% CI: 1.00–1.56, *p* = 0.05), dyslipidemia (HR: 1.19, 95% CI: 1.03–1.38, *p* = 0.02), and age (HR: 1.06, 95% CI: 1.04–1.09, *p* < 0.001), but not obesity, which was found significantly associated only in the univariable analysis (HR: 1.49, 95% CI: 1.24–1.79, *p* < 0.001). In contrast, alcohol consumption was protective, with an HR of 0.72 (95% CI: 0.58–0.88, *p* = 0.001).

**Table 2:** Independent association between rheumatoid arthritis (RA) and incidence of cardiovascular events in the whole GAZEL cohort

Variable	Univariable analysis		Multivariable analysis	
	HR (95% CI)	*p*	HR (95% CI)	*p*
Confirmed RA	**3.11 (1.17–8.32)**	**0.02**	**3.03 (1.13–8.11)**	**0.03**
Gender (male)	**2.37 (1.92–2.94)**	**< 0.001**	**1.85 (1.50–2.34)**	**< 0.001**
Tobacco consumption (≥ 20 PY)	**1.84 (1.58–2.15)**	**< 0.001**	**1.54 (1.31–1.80)**	**< 0.001**
High blood pressure	**1.78 (1.54–2.06)**	**< 0.001**	**1.51 (1.30–1.76)**	**< 0.001**
Diabetes	**1.72 (1.39–2.13)**	**< 0.001**	**1.25 (1.00–1.56)**	**0.05**
Dyslipidemia	**1.40 (1.21–1.62)**	**< 0.001**	**1.19 (1.03–1.38)**	**0.02**
Obesity (BMI ≥ 30)	**1.49 (1.24–1.79)**	**< 0.001**	1.17 (0.97**–**1.43)	0.11
Parental antecedent of myocardial infarction	1.17 (0.93**–**1.47)	0.18	1.11 (0.88**–**1.40)	0.37
Age (years)	**1.09 (1.07–1.12)**	**< 0.001**	**1.06 (1.04–1.09)**	**< 0.001**
Alcohol consumption	**0.83 (0.68–1.02)**	**0.07**	**0.72 (0.58–0.88)**	**0.001**

Analysis consisted in piecewise-exponential Poisson regression to assess longitudinal data from 2005 to 2014

### Identification of autoantibody-positive individuals

Plasma samples was available in 1618 subjects of the GAZEL cohort. As compared to the cohort without available plasma samples, subjects with RF or ACPA measurement had a similar prevalence of stroke and myocardial infarction. Also, CV risk factors were comparable between subjects with and without plasma sample except from hypertension (Table 1). With respect to the RA validation questionnaire, 9 RA patients were identified among the subjects with available plasma sample. All were RF or ACPA positive (ACPA+RF+: *n* = 8; ACPA+RF−: *n* = 1). Besides, 179 (11.1%) of the 1609 subjects without RA had either positive ACPA and RF (*N* = 8), ACPA only (*N* = 37), or RF only (*N* = 134). Among non-RA subjects, those with either positive ACPA or positive RF differed from the rest of the population only by a higher proportion of men (Table 3). During the observation period, 121 subjects without RA presented a new CV event: 12 had positive RF, 4 positive ACPA, and 105 were seronegative. Among the 9 RA patients, 3 had CV events.

**Table 3:** Characteristics of subjects without RA in the cohort with plasma sample available

Variable	Non-RA subjects with RF and/or ACPA+ (***N*** = 179)	Non-RA subjects without RF nor ACPA (***N*** = 1430)
Mean age (years)	60.8 ± 3.1	60.6 ± 3.3
Sex (% men)	160 (89.4%)	1164 (81.4%)^a^
Mean retirement age (years)	55.4 ± 2.6	55.4 ± 2.6
GAZEL 2005 questionnaire response rate	176 (98.3%)	1384 (96.8%)
Hypertension	32 (17.9%)	281 (19.7%)
Diabetes	9 (5%)	61 (4.3%)
Hyperlipidemia	40 (22.3%)	397 (27.8%)
Family history of MI	17 (9.5%)	99 (6.9%)
Body mass index	25.8 ± 3.1	25.6 ± 3.3
Smoking (% ≥ 20 pack-years)	36 (20.1%)	287 (20.1%)
Alcohol consumption	34 (18.9%)	267 (18.7%)
% ≥ to 21 glasses/week (men)		
% ≥ to 14 glasses/week (women)		
All events	5 (2.8%)	16 (1.1%)
Stroke	1 (0.6%)	5 (0.3%)
Myocardial infarction	4 (2.2%)	11 (0.8%)

Results are expressed as mean (standard deviation) or number (percentage)

^a^Significant difference between groups (*p* < 0.05)

### Association between RA-specific autoantibodies and CV events

The association between ACPA and CV risk was studied in non-RA subjects. No association was observed between the occurrence of CV events and ACPA positivity in those subjects (HR: 1.52, 95% CI: 0.47–4.84, *p* = 0.48, multivariate analysis) (Table 4). Similarly, no association was observed between RF positivity and CV events (HR: 1.15, 95% CI: 0.55–2.40, *p* = 0.70).

**Table 4:** Association of ACPA and/or RF positivity (without RA) with incident CV events in subjects with plasma sample available

Variable	Univariable analysis		Multivariable analysis	
	HR (95% CI)	*p*	HR (95% CI)	*p*
**Presence of ACPA**	2.07 (0.84–5.11)	0.12	1.52 (0.47–4.84)	0.48
**Presence of RF**	1.55 (0.49–4.90)	0.46	1.15 (0.55–2.40)	0.70
***Other factors***				
Gender (male)	1.86 (0.90–3.87)	0.09	1.20 (0.57–2.55)	0.63
Age (years)	**1.17 (1.09–1.26)**	**< 0.001**	**1.14 (1.06–1.23)**	**< 0.001**
High blood pressure	**2.19 (1.40–3.42)**	**< 0.001**	**1.87 (1.17–2.96)**	**0.008**
Diabetes	1.26 (0.55–2.90)	0.59	0.84 (0.35–2.01)	0.70
Dyslipidemia	1.57 (1.01–2.45)	0.50	1.42 (0.90–2.24)	0.13
Obesity (BMI ≥ 30)	1.14 (0.57–2.29)	0.71	0.87 (0.42–1.78)	0.70
Tobacco consumption (≥ 20 PY)	**2.21 (1.39–3.50)**	**< 0.001**	**1.87 (1.16–3.02)**	**0.01**
Alcohol consumption	0.91 (0.51–1.62)	0.75	0.78 (0.43–1.39)	0.39
Parental antecedent of myocardial infarction	1.01 (0.47–2.20)	0.97	0.85 (0.39–1.86)	0.69

This analysis only concerned the subjects with available blood sample for ACPA/RF testing, without RA (*N* = 1609)

## Discussion

In this study, we found that RA, as a clinical disease, but not RA-related autoimmunity was associated with CV events. It is known that RA is associated with two-fold increased risk for CV disease as compared to the general population [24–26]. While the overall CV risk in RA patients is based on traditional risk factors as well as autoimmunity related to RA, the increased risk due to RA is usually considered to be due to an increased inflammation and/or autoimmunity [24]. While systemic markers of inflammation have shown to be associated with a higher CV risk [27], other studies have also reported that CV risk is higher in RA patients with ACPA positivity [15–18], but such observation could be also explained by the correlation between ACPA positivity and RA severity [28], rather than by an independent association with ACPA. Hence, in the RA population, disentangling the effect of autoimmunity on CV risk from that of inflammation is difficult, if not impossible.

The fact that ACPA and RF positivity precedes RA and that some individuals are positive for RF or ACPA without even developing the disease allows to separately assess the role of RA-related autoimmunity and RA, as an inflammatory joint disease, on CV risk [4–6]. The analysis of GAZEL individuals that were positive for RF and/or ACPA permitted to directly evaluate the association between ACPA/RF and CV without the influence of arthritis. However, the analysis of these subjects was limited because of the rather low number of subjects with available blood sample, as compared to the whole cohort. Nevertheless, our analysis suggested that RA-related autoimmunity was not associated with an increased risk for CV disease, indicating that systemic inflammation is likely required for precipitating CV events. Under this hypothesis, it is conceivable that effector function of autoantibodies, i.e., Fc- mediated cytokine release, which translates asymptomatic autoimmunity to inflammatory disease might be critical for conveying CV risk [29].

In the GAZEL cohort, traditional risk factors such as male sex, age smoking, hypertension, hyperlipidemia, and diabetes mellitus were independently associated with CV events. Notably, the presence of RA also was significantly associated with CV disease with a HR of 3.0. The strength of the association between CV events and RA is reflected by the fact that the number of ascertained RA cases was rather low in this cohort but nonetheless this association was robust. This observation also supports the robustness of the lack of association between autoantibodies and CV risk as the numbers of autoantibody positive subjects was much higher than the one with RA. The overall low number of ascertained RA cases can be explained by the fact that participants of the GAZEL cohort were mostly males (> 70%). Considering a prevalence of RA of 0.5% in the French population [2], that only up to 1/3 of RA patients being males and that not all subjects with self-reported RA could be validated, the numbers of observed and established RA cases fits the numbers of expected RA cases.

Strength of this study includes the fact that the increased risk of CV events in RA patients as compared to controls was objectively confirmed and that the associations between RA and CV events on one hand, and autoantibodies and CV events in the other were assessed in the same cohort. Another strength is that the ascertainment of RA cases did not rely merely on self-reporting but was confirmed by experienced rheumatologist, using a dedicated questionnaire that was developed and validated for this purpose. A previous study assessed the association between autoantibodies and CV risk in a population-based cohort [30] and found that RF was a predictor of CV events. In this study, there was a higher number of subjects, but RA diagnosis was not confirmed by phone consultation, and CV comorbidities were analyzed only according to baseline characteristics. Concerning ACPA, only a part of the cohort was tested (*N* = 299), and the association between ACPA and CV events was not adjusted on rheumatic disease. Another study assessing the association between ACPA and RF and CV risk in non-RA patients found that the presence of autoantibodies was associated with CV risk increased in African American women, but the diagnosis of RA was again only based on self-reporting information [19]. A limitation of our study is due to the fact that plasma samples were not available in the entire GAZEL cohort and hence autoantibody data were only obtained in a fraction of the cohort. The fact that we found no significant association between RF and/or ACPA and CV events indicates however that there was no strong association between autoimmunity and CV risk, but does not formally allow to conclude to the absence of such association, considering the rather limited sample-size. On the other hand, subjects with plasma available did not essentially differ from the others with respect to demographic characteristics, CV risk factors and CV events. Furthermore, the robustness of a lack of association between autoantibodies and CV events is supported by the fact that positive association could be observed for RA, despite the fact that the number of RA cases was substantially lower than the number of subjects positively tested for autoantibodies. Finally, as the GAZEL cohort had a high ratio of men, and included subjects with a restricted age range, further studies including a larger number of subjects and a larger women representativeness will be needed to validate our results.

## Conclusion

These data suggest that CV risk in RA may rather be dependent on the inflammatory disease itself, while the mere presence of RA-related autoimmunity may not be associated, alone, with CV disease. Thus, the higher risk for CV events in autoantibody-positive RA may be related to a more severe and chronic course of the disease rather than direct effects of autoantibodies on the vessels. These data support the observations that effective control of inflammation may lower CV risk [31, 32].

## Supplementary Information

**Additional file 1.** Details on the specifically dedicated questionnaire for RA diagnosis confirmation and questionnaire translated from French.

## Data Availability

The data that support the findings of this study are available from Cohortes team of the Unit UMS 011 Paris University - Inserm - Versailles St-Quentin-Paris-Saclay University but restrictions apply to the availability of these data, which were used under license for the current study, and so are not publicly available. Data are however available from the authors upon reasonable request and with permission of Cohortes team of the Unit UMS 011 Paris University - Inserm - Versailles St-Quentin-Paris-Saclay University responsible for the GAZEL database management.

## Abbreviations

ACPA: Anti-citrullinated peptide antibodies
CV: Cardiovascular
DMARDs: Disease-modifying anti-rheumatic drugs
RA: Rheumatoid arthritis
RF: Rheumatoid factor
