# Supplementary material for: Rheumatoid arthritis, as a clinical disease, but not rheumatoid arthritis-associated autoimmunity, is linked to cardiovascular events
Source: Arthritis Res Ther. 2022 Feb 24;24:56. doi: 10.1186/s13075-022-02722-z (PMC8867622; doi:10.1186/s13075-022-02722-z)
Supplement: Supplementary file 1 — Additional file 1. Details on the specifically dedicated questionnaire for RA diagnosis confirmation and questionnaire translated from French. [file 13075_2022_2722_MOESM1_ESM.docx]

*Supplemental Material: Details on the specifically dedicated questionnaire for RA diagnosis confirmation and questionnaire translated from French*

Self-reported RA cases in the GAZEL cohort were validated by phone interview on the basis of a specific questionnaire. To evaluate its diagnostic performance, the dedicated questionnaire was applied through phone interview by a rheumatologist followed by independent chart review for validation of the diagnosis. This procedure was applied to a set of randomly selected volunteer patients having recently consulted n the Rheumatology department of Ambroise Paré Hospital. Of the 102 interviewed patients, 15 had confirmed RA and were all correctly identified by phone interview, yielding a sensitivity of 100%. Of the 71 confirmed non-RA patients, 8 were inappropriately diagnosed has having RA, yielding a specificity of 89%.

The questionnaire is presented below (translated from French).

| Gazel number |  |
| --- | --- |
| Birth date |  |
| Gender |  |
| Phone consultation date |  |
| Who made RA diagnosis?  -Rheumatologist?  -Other physician? |  |
| Can we contact your rheumatologist? |  |
| How old were you at diagnosis?  (Date at diagnosis) |  |
| Do you remember the date of first joint symptoms? (age at the beginning) |  |
| How long did symptoms last between the beginning and the diagnosis (persistence)? |  |
| Were your joints swollen? |  |
| Were your joints painful? |  |
| How many joints were swollen? |  |
| Could you precise which joints were swollen?  (describe joints by mean of keywords such as ‘groin’ instead of hip, ‘base of fingers’ instead of MCP, etcetera) |  |
| Was joint swelling confirmed by a physician? |  |
| How many joints were painful? |  |
| Could you describe which joints were painful (describe joints by mean of keywords such as ‘groin’ instead of hip, ‘base of fingers’ instead of MCP, etcetera) |  |
| On a scale of 0 to 10, where did you rate pain in joints? |  |
| Did you ever feel pain grasping the base of fingers or toes or when someone shakes your hands? |  |
| How long did you need to ‘stretch’ in the morning? (Stiffness) |  |
| Were you awakened at night because of joint pain? |  |
| Did you feel more exhausted than usual? Did you feel abnormal fatigue? |  |
| Do you or did you already have psoriasis?  (describe psoriasis) |  |
| Does anyone in your family, other than you, suffer from psoriasis?  (describe psoriasis) |  |
| Does anyone in your family, other than you, suffer from rheumatoid arthritis or inflammatory rheumatic diseases (first or second degree relative)? |  |
| Do you or did you ever have inflammation in your blood (sedimentation rate and /or CRP)? |  |
| Was rheumatoid factor analysis positive? If so, ask a result duplicate |  |
| Was anti-citrullinated peptide antibody analysis positive? If so, ask a result duplicate |  |
| Did you undergo joint X-rays? If so, ask for the report of most recent ones (hands, feet) |  |
| Were X-rays abnormal (erosions, joint space narrowing)?  Ask for the medical report |  |
| Can we retrieve your X-rays?  Can you read me or send me conclusions of the medical report? |  |
| Which treatments did you received?  -Corticosteroids?  -Methotrexate?  -Leflunomide?  -Hydroxychloroquine?  -Sulfasalazine?  -Gold salts?  -Nonsteroidal anti-inflammatory drugs?  -Treatments with subcutaneous injections?  -Which one?  -Treatment with intraveinous infusions?  -Which one? |  |
| -Do you have artificial joints?  -If so, which ones?  -Do you have arthrodesis?  If so, which joints? |  |
| Which treatment do you actually receive? |  |
| On a scale of 0 to 10, how do you currently feel? |  |
| On a scale of 0 to 10, where do you currently rate joint pain? |  |
| How long do you currently need to ‘stretch’ in the morning? (Stiffness) |  |
| How many joints are currently painful? |  |
| How many joints are currently swollen? |  |
